# Impartial Third-Party Interventions in Captive Chimpanzees: A Reflection of Community Concern

**DOI:** 10.1371/journal.pone.0032494

**Published:** 2012-03-07

**Authors:** Claudia Rudolf von Rohr, Sonja E. Koski, Judith M. Burkart, Clare Caws, Orlaith N. Fraser, Angela Ziltener, Carel P. van Schaik

**Affiliations:** 1 Anthropological Institute & Museum, University of Zurich, Zurich, Switzerland; 2 University Research Priority Program in Ethics, University of Zurich, Zurich, Switzerland; 3 School of Natural Sciences and Psychology, John Moores University, Liverpool, United Kingdom; 4 Department of Cognitive Biology, University of Vienna, Vienna, Austria; University of Plymouth, United Kingdom

## Abstract

Because conflicts among social group members are inevitable, their management is crucial for group stability. The rarest and most interesting form of conflict management is policing, i.e., *impartial interventions by bystanders*, which is of considerable interest due to its potentially moral nature. Here, we provide descriptive and quantitative data on policing in captive chimpanzees. First, we report on a high rate of policing in one captive group characterized by recently introduced females and a rank reversal between two males. We explored the influence of various factors on the occurrence of policing. The results show that only the alpha and beta males acted as arbitrators using manifold tactics to control conflicts, and that their interventions strongly depended on conflict complexity. Secondly, we compared the policing patterns in three other captive chimpanzee groups. We found that although rare, policing was more prevalent at times of increased social instability, both high-ranking males and females performed policing, and conflicts of all sex-dyad combinations were policed. These results suggest that the primary function of policing is to increase group stability. It may thus reflect prosocial behaviour based upon “community concern.” However, policing remains a rare behaviour and more data are needed to test the generality of this hypothesis.

## Introduction

Group living and hence sociality is a widespread phenomenon among animals. Because groups are often composed of individuals of different age, sex and relatedness, conflicts arise concerning reproduction or access to resources. Such conflicts may dramatically increase when groups are perturbed from the outside or undergo changes in composition through dispersal or immigration. Conflicts may disrupt group stability, so if individual fitness is dependent on group stability [1], evolution should favour mechanisms that decrease disruption [2], [3].

Researchers have identified several mechanisms through which social animals, especially nonhuman primates, manage conflicts, including dominance [4], reconciliation [3], bystander affiliation to recipients and/or initiators of aggression [5], [6], [7], [8], [9], mediation [10], punishment [11], [12] and policing [1], [13]. The focus of this paper is on events of policing, which we define as *impartial* interventions by third parties in ongoing conflicts. Being impartial, these interventions never include aggression directed specifically at one of the contestants. Such policing is different from the common partial bystander involvement in conflicts, which involves agonistic support of one of the contestants. It is also different from punishment [11], which concerns aggression directed specifically at the wrongdoer. To emphasize the impartiality of the performers of policing, we call them “arbitrators”.

Policing has been reported in chimpanzees [14], bonobos [15], mountain gorillas [16], [17], [18] and in captive Bornean orang-utans [19], [20]. Other species include the golden monkeys [21], hamadryas baboons [22], and several macaques species such as Barbary (A. Bissonnette; unpublished data), rhesus [23], [24], Japanese [25], pigtailed [13], [26] and Tonkean macaques [27].

Policing is risky because it requires approaching two or more fighting contestants, which may lead to becoming the recipient of aggression [13]. Additionally, arbitrators may incur energy and opportunity costs. To be favoured by natural selection, therefore, policing should bring fitness benefits for the arbitrator. However, the impartiality makes it difficult to recognize such direct fitness benefits.

Various hypotheses have been proposed for the function of policing in nonhuman primates. However, because policing is a relatively rare behaviour among primates, the cross-species data thus far has not consistently supported any functional hypothesis. In this paper, we discuss the proposed hypotheses and develop predictions for policing in chimpanzees, where conflicts often arise among females over access to food [28] or among males over access to females [29] and may result in severe dyadic or even polyadic agonistic interactions [10].

The most popular hypothesis claims that policing brings only indirect benefits to the arbitrator, because it serves to increase group stability [30] by reducing the number of conflicts [13], [30] and by allowing all individuals build up larger and more diverse social networks [1]. Increased group stability may indirectly increase the arbitrator's fitness via the reproductive benefits of living in a stable network of beneficial relationships [26]. In terms of proximate causation [31], [32], policing might be motivated by a concern about the conflicts of others and thus might reflect a basic “community concern” [33]. As a result, policing has been seen as a precursor of human morality [33], [34], and therefore may inform of its evolution.

If the increase of group stability is the primary function of policing, it should occur most often when group stability is weakened (see Table 1 for predictions). Group instability may arise due to major relationship changes, such as rank reversals near the top of the hierarchy, death of an important social player, or immigration/emigration. In such unstable circumstances, relationships are easily damaged and thus group stability is at stake. First, the *group stability hypothesi*s predicts that arbitrators are high-ranking individuals because high-ranking individuals have the power to effectively stop aggression [13] and simultaneously have a lower risk of receiving aggression upon approaching combatants. Second, it predicts that arbitrators can be both male and female because both sexes have a stake in group stability. Social instability in a group constitutes a major stress factor for all group members as it has negative influences on social relationships and hence various social interactions such as grooming, play and physical contact [1] and also individual health [35], [36]. Hence, all group members have a stake in group stability but not all individuals possess enough social power to control group stability. Third, policing should occur in conflicts that are most likely to disrupt relationships in a group, in particular those involving intensive aggression involving multiple individuals. Finally, because of the primacy of conflict intensity, policing should not be limited to particular sex-dyad combinations.

**Table 1:** The proposed hypotheses and their predictions for the function of impartial interventions in chimpanzees.

Hypotheses	Conditions of policing	Who are the arbitrators?	Policed sex-dyad combinations
*The group stability hypothesis*	Presence of social instability	High-ranking individuals of both sexes	Conflicts of all sex-dyad combinations with high escalation potential
*The assurance of dominance hypothesis*	Decrease in dominance possible (e.g. old age, social climber)	High-ranking males	Male-male
*The assurance of sexual benefits hypothesis*	Immigration of females	High-ranking males	Female-female

Whereas the *group stability hypothesis* may indirectly increase the arbitrator's fitness, alternative hypotheses propose direct benefits. Thus, a second hypothesis assumes that policing helps high-ranking individuals to assert their social interests [37], [38], [39]. This *assurance of dominance hypothesis* is supported in fallow deer (*Dama dama*), where impartial interventions are a male strategy to control other males' social advance in the hierarchy [40], [41]. The hypothesis predicts for chimpanzees that arbitrators are high-ranking males, and that policing occurs only in conflicts among direct social competitors, i.e. in male-male conflicts. It does not expect the same pattern for females because chimpanzee females tend not to fight over rank.

A third hypothesis is that policing functions to assure sexual benefits. It has been argued that in species where females transfer between groups, males may police female-female conflicts to discourage females from emigrating, and so preserve reproductive payoffs for the policing males [39]. In addition, policing may reduce stress faced by females due to aggressive conflicts. In chimpanzees, as in other male-philopatric species, immigrant females often meet aggressive resistance by resident females [42], [43], [44], [45] and male policing could make immigrants more likely to stay in the group. Indeed, by policing such conflicts males may also be able to establish and preserve valuable relationships with the females without damaging their relationship with either opponent [39], [46]. This *sexual benefits hypothesis* predicts that policing is always performed by males, who intervene only in female-female conflicts.

Finally, policing may be directly self-serving in preventing escalated aggression from “spilling over” to affect the policing individual. In this case, we expect it to be performed by individuals with the highest likelihood of being targets of aggression, i.e. low-ranking individuals. However, given the potential risk of intervening in agonistic interactions, the existing literature on policing in various species does not support this *protection from aggression* hypothesis, as arbitrators appear to be predominantly high-ranking individuals [13], [47], [48]. Therefore, we will not consider this hypothesis any further.

The aim of this study is to test the predictions of the three hypotheses to identify the function of policing in chimpanzees (see Tab. 1). The predictions focus on the identity of the arbitrators, the kinds of dyads in which interventions are expected, and the role of social instability. First, we report a detailed study of a captive group of chimpanzees (Gossau) with a high level of social instability and a high rate of policing, which allowed us to directly test the aforementioned hypotheses. This represents the first systematic study of chimpanzee policing, which so far has been reported on a rather anecdotal level in the literature [14], [47], [49]. Second, we compare the policing patterns found in Gossau with three other captive chimpanzee groups (Basel, Chester, Arnhem). The comparison relies on data collected earlier for other research purposes. Therefore, the policing data are extracted from the records *post hoc*. This comparison allowed us to assess the generality of the findings of the first part regarding the identity of arbitrators, sex-dyad combinations of policed conflicts and, qualitatively, the social conditions in which policing occurred.

## Methods

### Ethics Statement

The main study conformed to all regulations regarding the ethical treatment of animals and was formally approved by the veterinary office of St. Gallen, Switzerland. All zoos mentioned in this paper belong to the EAZA (European Association of Zoo and Aquaria) and therefore comply with their welfare requirements. Furthermore, they follow the guidelines contained in the Weatherall Report for the use of nonhuman primates in research. Animals were never separated from each other for the purpose of the studies mentioned here. We did not induce any aggression and all data were collected observationally on behaviour that occurred spontaneously.

### Part I

#### Study Subjects and Housing

Data were collected from a captive chimpanzee (*Pan troglodytes*) group housed in an indoor-outdoor facility (indoor facility: 2×150 m^2^, outdoor facility: 2×450 m^2^) at the Abenteuerland Walter Zoo (Gossau), Switzerland. The group consisted of 11, mainly captive-born, individuals, two adult males and one adolescent male, six adult females and two adolescent females. This reflects the group composition in the beginning of February 2007, after the introduction of three new adult females. For further details on the individuals, see supporting information (SI) Table S1. Shortly after data collection had started, three adult females from another group were introduced into the main study group. In addition, there was a rank reversal between two males taking place. These events resulted in social instability during the study period (see *Methods*: *Rank Hierarchy and Stability* for details).

#### Data Collection and Analysis

Data were collected on the whole group over 22 hours in February 2007 by AZ and over 564 hours from May 2007–November 2008 by CRvR, using all occurrence and scan sampling [50]. Observations took place throughout the day while the chimpanzees were in the indoor or outdoor enclosures, and included all occurrences of social interactions such as affiliation, aggressive conflicts, dominance and sexual interactions, as well as third-party behaviour. The ethogram used in this study was based on van Hooff [51]. For further details on the ethogram, see Table S2.

We recorded subordination signals to determine an individual's dominance rank. The chimpanzee's most obvious subordination signal is the pant grunt. This vocalisation is always directed up the hierarchy [52], [53]. Other subordination signals included in the determination of dominance hierarchy were avoidance, fleeing and presenting behaviour. In total, we collected 1299 subordination signals. The Elo-rating method [54], [55], ran in the statistical environment R (Version 2.12.1) [56], was used to investigate and visualize individual dominance rank and rank instability. For each individual we chose a starting value of 1000 and a constant *k* = 200 was used. This allowed us to establish the hierarchy among males and females and to detect rank instability.

Factors influencing the occurrence of policing were analyzed using generalized linear mixed effects models (GLMM) [57]. We chose eight factors that we thought as most likely to be important in influencing a chimpanzee's intervention behaviour. For an overview and descriptions of the fixed and random factor(s), see Table S3. Conflict characteristics (directionality, intensity, complexity), identity of conflict participants (maternal kin, “friend”, immigrant female), class of conflict (sex-dyad combination) and identity of arbitrator (male) were entered as fixed factors. Paternal kin was not included as a factor in the GLMM because maternal relatedness results in stronger bonds than paternal relatedness [58], [59] and because of a lack of strong evidence of paternal kin identification mechanisms in primates, including chimpanzees [60]. The date of data collection was entered as a random factor. GLMMs were fitted with lme4 [61] using the glmer procedure in the statistical environment R (Version 2.12.1) [56] with binomial error structure.

To assess “friendships” among individuals, a social proximity scan including social grooming was performed every 5 minutes, recording each individual's distance to each other individual if the individuals were not moving and all social grooming. We collected 96 scan samples in February 2007 and 3472 scan samples from May 2007–November 2008. Social grooming and close proximity were used to calculate a composite index of sociality (CIS) for each dyad (*N = 55* dyads) [58]. Grooming and proximity are commonly used to proxy “friendship” or close social bonds in primate groups [62]. In chimpanzees, grooming and proximity reflect the value dimension of social relationships. While social relationships also encompass other relationship quality dimensions, namely compatibility and security [63], we did not include them in the analyses for two reasons. First, security measures were not recorded in data collection, and second, value was considered as a more directly important variable than compatibility (“friendliness”) in a decision of whether or not to police a conflict. The dyads for which CIS was below 1.5 were classified as having a weak social bond, or to be “non-friends” [56]. The dyads with CIS equal to or above 1.5 (18.2% of all dyads) were classified as having a strong social bond and hence were classified as “friends”.

#### Rank Hierarchy and Stability

**Figure 1:**
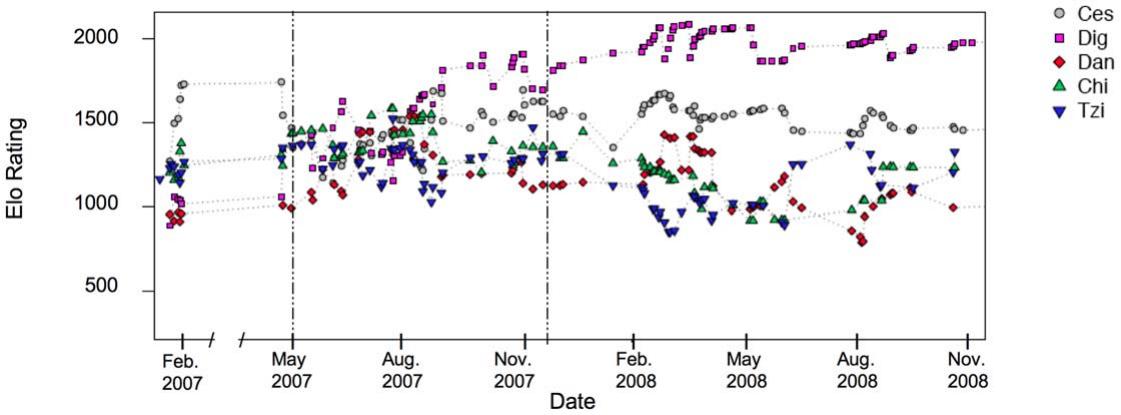
Elo-ratings of the highest-ranking individuals of the study group in Gossau for the time range of February 2007 until the end of the study in November 2008. Each line represents an individual. Each symbol represents an Elo-rating after they were updated following an interaction of the depicted individual. Dotted lines indicate the time range of rank instabilities in the study group. Note: No data collection was performed in March and April 2007.

For a better overview, we only plot the most dominant individuals (Ces, Dig, Dan, Chi, Tzi), see Figure 1. We found that by the end of February 2007 – about one month after the introduction of the three new females – Ces occupied the alpha position, just as he had been before the introduction and for several years before the current study (zoo staff, pers. comm.). The youngest male Dig (15 yrs) had begun to challenge the then alpha male Ces before the introduction (zoo staff, pers. comm.), but was not yet successful in defeating Ces. In May 2007, Ces was observed to submit formally to Dig, indicating that a rank reversal was in progress between these two males and that it must have started between February and May 2007. The two immigrants females (Chi and Tzi) aimed for a high position in the hierarchy and were able to establish themselves in high rank positions. By the end of November 2007, Dig had established himself in the alpha position and remained there until the end of data collection in November 2008. The immigrant females ranked at that time right below Ces (see Fig. 1). Figure 1 visualizes the rank instability during the first few months after the introduction among the highest-ranking individuals.

### Part II

#### Study Subjects and Data Collection

We compare the policing pattern found in Gossau with data from three other captive chimpanzee groups: Basel, Chester and Arnhem (Burgers) Zoos. Except in Basel, the data on impartial interventions were extracted post-hoc from earlier studies for the present analysis, and only physical interventions were included (physical nonaggressive but impartial separation, or run-through). Table 2 provides an overview of policing events of all groups. Basel Zoo, Switzerland, houses 10 chimpanzees (two adult males, four adult females, three infants and one juvenile female) in a well-established social group. The operational definition of policing was identical to the main study. Conflicts were recorded ad libitum during 3 months (March–May 2009) by CRvR. During the study, male ranks were unstable. The alpha male was very old, and the other younger male had started to challenge him. The males did not associate with or groom each other. Females were equally submissive to both males.

**Table 2:** Overview of the impartial interventions of all groups.

	Social instability	N Conflicts	Policing (%)	Sex-dyad combination of policed conflicts	Arbitrators
**Gossau**	*Yes*	*438*	*15.75*	*All sex-dyad combinations*	*High-ranking males*
**Basel**	*Yes*	*66*	*6*	*Female-female*	*High-ranking males*
**Chester (1)**	*Yes*	*4000*	*0.2*	*Female-female, male-male*	*High-ranking females, large mid-ranking male*
**Chester (2)**	*No*	*256*	*0*	*n/a*	*n/a*
**Arnhem (1)**	*Yes*	*376*	*2.7*	*All sex-dyad combinations*	*High-ranking males and females*
**Arnhem (2)**	*No*	*365*	*0.8*	*Female-female, male-male*	*High-ranking males and females, a young male (social climber)*

Chester Zoo, UK, houses a well-established social group whose size has varied from 26 to 32 chimpanzees (17 adult females, five adult males and four to 10 infants and juveniles) over the years. Here we assess data collected in two phases. The first phase “Chester (1)” spans three years (January 2000–January 2003), during which conflicts were observed ad libitum by CC. This period was characterized by an initially unstable male dominance hierarchy, which however stabilized during the course of the study. However, we do not have data to formally assess the male dominance hierarchy, so the instability is based on qualitative assessment of the group dynamics. The second phase “Chester (2)” consists of a detailed study on within-group aggression during 2005 and 2006, including 18 months of ad libitum sampling of conflicts by ONF. This period was highly stable, as reflected by the fact that hardly any formal signals of subordinance were given (ONF, unpublished data).

Arnhem (Burgers Zoo), the Netherlands, houses chimpanzees in a well-established social group of 23–35 individuals (three to five adult males, 14–18 adult females, zero to six adolescents, four to eight infants and juveniles). Conflicts were observed ad libitum over the course of three years (June 2002–August 2005) by SEK and her students. Although the study was nearly continuous, the data are here divided into two phases “Arnhem (1)” and “Arnhem (2)” due to a difference in group stability. “Arnhem (1)” was characterized by instability, caused by the death of an adult female and the removal of an adult male, who shared the alpha position with his brother. Following his removal, a young male, previously fourth in rank, took over the alpha position (all these events occurred between December 2002 and January 2003). During the subsequent months, until August 2003, male rank was unstable, as indicated by inconsistent submission signals by both males and females. “Arnhem (2)” combines observations prior to the death and rank reversal (June–October 2002) and after the rank had stabilized (August 2003–August 2005). During these periods male rank was stable and no incidents of social instability occurred.

## Results

### Part I

#### Conflicts, Arbitrators and Frequency of Policing

We observed 438 conflicts of which 202 (46.1%) were female-female, 186 (42.5%) male-female and 50 (11.4%) male-male. 26 (5.9%) conflicts were polyadic. Conflicts between resident and immigrant females occurred regularly and accounted for the largest number of conflicts (173 cases; 39.5%). Policing occurred in 69 conflicts (15,75%) and consisted of 38 female-female, 27 male-female and 4 male-male conflicts. This did not differ from the overall distribution of conflicts among sex-classes (*χ* ^2^ (2) = 2.94, *P* = 0.23). On eight occasions, the two males performed impartial interventions simultaneously.

Policing was exclusively restricted to two males, Ces (*N = 44*) and Dig (*N = 25*). The third male Dan never engaged in this behaviour. Notably, when Ces was the alpha male, he intervened in 11 conflicts, whereas Dig only started to police conflicts in May 2007. Of the 69 policing events we observed during data collection, Dig intervened in 10 (40%) female-female conflicts, in 12 (48%) male-female conflicts and in three (12%) male-male conflicts. Ces performed in total 44 impartial interventions, of which 28 (63.6%) were in female-female conflicts, 15 (34.1%) were in male-female conflicts and one (2.3%) was in a male-male conflict.

#### Tactics and Success of Policing

Policing tactics included passive as well as active interventions. Passive interventions were called attendance and defined as a third party approaching a conflict in a directed manner until within a short distance showing no other behaviour [13]. This type of intervention was observed in 31 cases (44.93%). Active interventions ranged from threatening both antagonists simultaneously in two cases (2.9%), to interpositions in four cases (5.8%) and “running through” the conflict in 26 cases (37.68%). Interpositions were defined as a third party standing between the antagonists and “running through” as a third party running between the antagonists. In six cases (8.7%), one male threatened both conflict participants simultaneously while the other “ran through” the conflict.

We considered policing successful if it immediately terminated a conflict. Of the 69 impartial interventions, 60 (86.96%) were successful and nine (13.04%) were not. All eight impartial interventions that were performed simultaneously by the two males were successful. The number of times policing was unsuccessful per individual was four and five, respectively. The males received only once aggression in response to policing, indicating that combatants may have regarded arbitrators as authorities.

#### Determinants of Policing

**Figure 2:**
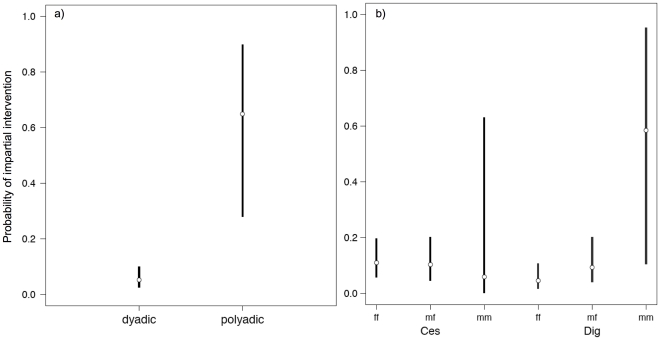
Plots showing the 95% credible intervals for a) complexity of a conflict (dyadic vs. polyadic) and for b) the interaction between class of conflict (sex-dyad combination) and identity of arbitrator (male).

To investigate whether the two arbitrators followed different strategies when intervening, we included in the GLMMs all fixed factors and all two-way interactions with arbitrator (male). Since the model with all two-way interactions with male did not converge, we added only one interaction at a time and used the Akaike's Information Criterion [64] to select the model with the best fit to the data [65]. Thereby, only interactions that reduced the AIC by >2 units were included [66]. The selected model contained all fixed factors and the class x male interaction and had an AIC of 328.3. The AICs of the excluded models ranged between 334.9 and 332.9 and were significantly different from the selected model (Log-Likelihood Ratio Tests: ^Δ^ *χ* ^2^(1) = 8.589, *P* = 0.003; ^Δ^ *χ* ^2^(2) = 8.631 *P* = 0.01, respectively). The overall significance of the selected model against the null model, including only the intercept and the random factor was also tested [65]. The null model (AIC = 337.68) was significantly different from the selected model (Log-Likelihood Ratio Test: ^Δ^ *χ* ^2^(12) = 33.387, *P*<0.001). Therefore, the selected model explained significantly more variance in the data than the excluded models as well as the null model. Table 3 shows the parameter estimates of the selected model. We present the Bayesian credible intervals [67] of the significant parameter estimates in addition to the z-test, since the z-test is only an approximation and could therefore be misleading [68]. The 95% credible intervals show that polyadic conflicts had a significantly higher probability of attracting impartial intervention than dyadic conflicts (see Fig. 2a). Furthermore, the males differed to some extent regarding which class of conflict they preferred to intervene in. The younger male Dig showed a trend to intervene preferentially in male-male conflicts compared to the other classes (see Fig. 2b).

**Table 3:** Factors in the selected model (GLMM) explaining the occurrence of impartial interventions in arbitrators.

Fixed factors	*Estimate*	*Std. Error*	*z value*	*P (>|z|)*
(Intercept)	−2.896	1.272	−2.277	0.023*
Directionality unidirec	−0.471	0.518	−0.909	0.364
Intensity low	−0.125	0.454	−0.274	0.784
Complexity polyadic	4.627	0.997	4.641	3.47e-06***
Maternal kin yes	−0.348	1.141	−0.305	0.760
Friend no	0.957	1.056	0.906	0.365
Friend yes	0.388	0.898	0.432	0.666
Immigrant female yes	−0.265	0.542	−0.489	0.624
Class mf	0.373	0.659	0.567	0.571
Class mm	−4.299	2.551	−1.686	0.092.
Male Dig	−0.941	0.642	−1.466	0.143
Class mf×male Dig	0.339	0.842	0.402	0.687
Class mm×male Dig	8.264	3.058	2.703	0.007**

Significance codes:

“***” 0.001.

“**” 0.01.

“*” 0.05.

“.” 0.1.

In sum, two of the three males performed policing and were effective in ending the conflicts. They policed conflicts among all sex-dyad combinations, although one male tended to intervene more often in male-male conflicts, and policing was most likely executed when a conflict involved multiple combatants.

These results made it possible to refute the assurance of sexual benefits hypothesis, but did not allow us to distinguish between the group stability hypothesis and the assurance of dominance hypothesis, as one male tended to intervene in male-male conflicts. Therefore, we followed up the study with a cross-zoo comparison on policing behaviour.

### Part II

Table 2 summarizes the results of the comparison of the groups. The overall pattern is mostly consistent with the group stability hypothesis, although there were differences among the zoos in policing patterns. First, only the group stability hypothesis predicts that high-ranking individuals of both sexes should engage in policing. In Basel, all policing was exclusively performed by the alpha and beta male, which were the two only adult males in this group. In Chester, a mid-ranking male, who was the largest male in the group, and two high-ranking females were responsible for the policing. In Arnhem, high-ranking males and females performed all but one of the interventions. In 10 of 13 cases the arbitrator was the alpha, beta or gamma male, and in two cases it was a high-ranking female. In one case the arbitrator was a lower ranking male, who was rapidly rising in rank.

Second, only the group-stability hypothesis predicts that arbitrators intervene in interactions, regardless of sex-dyads combinations, rather than exclusively in male-male (assurance of dominance hypothesis) or female-female (sexual benefits hypothesis) dyads. In Basel, all policed conflicts were dyadic female-female conflicts (*N* of all conflicts: female-female: *34*; male-female: *23*; male-male: *one*; adult-immature: *eight*). In Chester, of the seven policed conflicts, six were female-female and one was male-male. In Arnhem, five female-female, six male-female and two male-male conflicts were policed. In sum, across these three groups, agonistic interactions involving both sexes and all three possible combinations were subject to interventions by arbitrators, and arbitrators were males and females, a pattern most consistent with the group stability hypothesis.

The group stability hypothesis also predicts that intensive or otherwise escalation-prone conflicts are policed. This prediction was supported in Gossau and Basel. In Chester, most policing occurred in moderate to severely aggressive dyadic conflicts. In Arnhem, eight out of 13 policed conflicts were severely aggressive, four of which polyadic, and the remaining five were dyadic conflicts with mild physical aggression. Thus, the majority, but not all, of the policed conflicts involved severe aggression, but there was no consistent bias towards polyadic conflicts as found in Gossau.

Finally, the degree of social instability is predicted to increase occurrence of policing. As the degree of social instability could not be assessed quantitatively, we estimated the presence and absence of instability in the three zoos qualitatively, based on aggression frequency and patterns and inconsistency of submissive signals indicating instability of male dominance hierarchy. Policing was relatively more common in Arnhem (1) than in Arnhem (2). Similarly, it was relatively more common in Chester (1) than in Chester (2). Finally, policing was remarkably frequent in Basel, reaching almost the same values as in Gossau. Overall, therefore, policing was most likely at times of social instability. This further corroborates the group stability hypothesis.

## Discussion

We explored impartial interventions, i.e. policing, as a conflict management mechanism in chimpanzees. We addressed three hypotheses for the function of policing in chimpanzees: a group-stabilizing function, consistent with an expression of “community concern” by the arbitrators; male assurance of dominance by prohibiting rise of social competitors; and male assurance of sexual benefits by alleviating female-female aggression and simultaneously improving own relationship with females.

In the main study, we found that only adult, high-ranking males performed policing and they policed conflicts of all sex-dyad combinations. The primary predictor of policing was conflict complexity, in that polyadic conflicts were policed more often than dyadic ones. The occurrence of policing across all sex-dyad combinations does not support the assurance of dominance hypothesis or the assurance of sexual benefits hypotheses, but is consistent with the group stability hypothesis. Moreover, the high prevalence of policing coincided with social instability in the group, i.e. the introduction of three adult females and a rank reversal between the two top-ranking males. Thus, social relationships were unstable and easily disturbed. Policing as a group stabilizer may have prevented conflicts from escalating, thereby preventing further disruption of group stability.

However, as all policing in Gossau was performed by adult males, and one of them showed a tendency to police male-male conflicts, we could not distinguish between the group stability hypothesis and the assurance of dominance hypothesis. Therefore, we conducted a broader evaluation of the hypotheses by combining policing data from three other captive groups. The comparative data added support for the group stability hypothesis. High-ranking individuals of both sexes performed the vast majority of policing, and they intervened in conflicts among all sex-dyad combinations. Moreover, although policing was rare overall, policing was more likely during times of social instability. These patterns are consistent with the hypothesis of a group-stabilizing function.

Available reports from wild chimpanzees give further support to this hypothesis. In both Mahale and Gombe National Parks [47], [48], [69], arbitrators were high-ranking individuals of both sexes [28], conflicts of all sex-dyad combinations were policed and, at least in Mahale, occurred at times of social instability. Altogether, it appears plausible that the main function of policing in chimpanzees is to stabilize group dynamics.

The data did not support the two alternatives proposing a direct fitness benefit to the interveners. Moreover, an alternative possibility to explain policing, namely that arbitrators merely dislike noisy disturbances and take action to stop them [12], [52], also seems unlikely because policing is relatively rare while noisy conflicts occur frequently.

The results stressed the importance of social power in effective policing. In Gossau, arbitrators exhibited a high success rate of policing (86.96%) and almost never received aggression in response to their behaviour. The near-absence of aggression towards arbitrators is not surprising given their high social ranks. In the other zoos too, the arbitrators were high-ranking in nearly all cases, and in the only two exceptions (once in Chester and once in Arnhem) they were nevertheless individuals of potential social importance. The fact that mostly high ranking individuals engaged in policing is consistent with the theoretical models of Frank [30], [70], [71] and the empirical work in pigtailed macaques of Flack et al. [13], in which heterogeneities in power lead to heterogeneities in the tendency to police. The more powerful an individual is, the more effective it is in controlling a conflict and this at a low cost [72].

An interesting exception to this pattern was seen in Gossau, where the introduced females, despite being high-ranking and large-bodied, did not engage in policing. We think that this might be explained by the fact that as newcomers, they were not yet fully accepted by the others and thus did not hold the necessary social power. Alternatively, the immigrant females may have lacked the motivation to police, if they did not yet regard the group as their “own”. Another limiting factor might have been the fact these females conceived soon after the introduction and subsequently carried vulnerable infants.

When policing, arbitrators in Gossau used different tactics, mainly preferring to literally “run through” the conflict thereby separating the combatants, or to attend the conflict (see *Results: Tactics of Policing* for definition). For the latter tactic to be effective, arbitrators need to be perceived by all combatants as being more powerful than themselves. Similarly, various policing behaviours were seen in Arnhem and Chester. However, we could reliably extract only policing events that involved clear physical involvement. Thus, the overall prevalence may have been underestimated by excluding the passive attendance-type of interventions. However, we think these conclusions are not affected by this possible bias, because passive interventions are likely to have been done by high-ranking individuals, as in Gossau.

Policing occurs only rarely in chimpanzees, which is probably why its function has remained elusive to researchers. We hypothesize that several preconditions are necessary for impartial interventions to occur regularly. First, conflicts that occur in a group must have the potential to endanger group stability. This is probably not the limiting factor in chimpanzees, where intensive, polyadic conflicts occur regularly. Second, relationship stability in a group must be challenged, leading to increased likelihood of disruption by conflicts. This aspect may explain why policing is so rare. Several effective, mainly dyadic, conflict management mechanisms exist in the chimpanzee behavioural repertoire, which may suffice maintaining group stability under normal conditions. However, rank reversals among the highest-ranking individuals, or significant demographic changes such as the removal or addition of adults may promote conditions in which policing emerges. Third, a group needs to include individuals that possess sufficient authority and power to successfully control conflicts. Gaining authority may be associated with long-term membership and high rank in the group, and given chimpanzee socio-ecology, lack of authoritative individuals is probably not the explanation for scarcity of policing in chimpanzees. However, personality may influence the individual tendency to engage in policing. High-ranking individuals are not necessarily all equally likely to be involved in group activities [73]. Future studies should explore whether chimpanzees change their policing strategy during their lifetime as they rise or fall in rank, whether it is dependent on the group composition, and whether it is determined by individual personality of the arbitrators.

Increase of group stability by active policing may be rooted in a basic “community concern”, i.e. the motivation to maintain stable, harmonious dynamics in a group [33]. Although there may also be additional, self-serving benefits at the proximate level (e.g. females may prefer males that engage in policing as mates and allies [14]), group-stabilizing policing may be driven by a pacifying motivation and as such, can be considered as prosocial behaviour.

In humans, community concern is expressed in its highest degree and can be seen as the very foundation of human morality and indeed social norms. Thus, in humans, as in chimpanzees, community concern may constitute one of the proximate mechanisms for conflict management, which likely is independent of its ultimate goal of group stability, which in turn increases fitness of group members. Thus, from a proximate perspective, policing behaviour may be genuinely prosocial in that arbitrators perform it without self-serving motives [32], [74].

Theoretical and empirical studies on the emergence of human large-scale cooperation have shown that one of the important mechanisms is the existence of punishment, either directly or through externalized “pool-punishment” forces [75]. Institutionalized pool-punishing by law enforcement effectively maintains cooperation through social norms [76]. Nonhuman primates, despite the existence of small-scale cooperation, social learning and potentially proto-normative behaviour [77], do not exert direct or pool-punishment. Neither do most small-scale human foragers [78]. Thus, the policing as a conflict management mechanism seen among nonhuman primates might be a precursor for large-scale “police forces” that maintain normative behaviour in large-scale human societies.

In conclusion, we found that although policing behaviour is overall rare, it occurs in several chimpanzee groups. Its frequency appears to increase at times of social instability. High-ranking individuals of both sexes police conflicts, and all sex-dyad combinations of conflicts are policed. The higher the conflict's disruption potential (i.e. polyadic and/or severe aggression), the more likely it is policed. This was especially shown in the main study, in which we could assess the occurrence of policing with a predictive model. We hypothesized that high rates of policing are due to considerable social instability, which may disrupt the group's social structure. Policing was highly effective in stopping conflicts. These results suggest that the main function of policing is to maintain the group's social stability. This behaviour may reflect arbitrator's pacifying motivation in the form of a basic “community concern”.

## Supporting Information

Table S1 Details on the group composition after the introduction of the three new females. Individuals are ranked according to age within their sex. Arrows indicate older offspring. ^†^ Females, who gave birth to new offspring within the data collection period. Their offspring was excluded from data collection and is therefore not listed here.
(DOC)

Table S2 Ethogram used for observations during this study.
(DOC)

Table S3 Factors used in the generalized linear mixed models (GLMMs) to explain impartial interventions. ^a^ *Directionality*: scored as bidirectional if both participants engaged in aggressive behaviour and as unidirectional if all aggressive behaviour was directed toward the initial recipient. ^b^ *Intensity*: scored on a two-level scale: low intensity  =  conflict involved aggression without physical contact; high intensity = conflict involved physical aggression. ^c^ *Complexity*: scored as dyadic if one individual threatened or aggressed a second individual and as polyadic if more than two individuals were involved in the conflict.
(DOC)
