# Supplementary material for: Impartial Third-Party Interventions in Captive Chimpanzees: A Reflection of Community Concern
Source: PLoS One. 2012 Mar 7;7(3):e32494. doi: 10.1371/journal.pone.0032494 (PMC3296710; doi:10.1371/journal.pone.0032494)
Supplement: Table S1 — Details on the group composition after the introduction of the three new females. Individuals are ranked according to age within their sex. Arrows indicate older offspring. † Females, who gave birth to new offspring within the data collection period. Their offspring was excluded from data collection and is therefore not listed here. (DOC) [file pone.0032494.s001.doc]

| **Individuals** | **ID** | **Sex** | **Age class** | **Age** | **Residence Status** |
| --- | --- | --- | --- | --- | --- |
| Cess | Ces | M | Adult | 31 | Resident |
| Dandy | Dan | M | Adult | 23 | Resident |
| Digit | Dig | M | Adolescent | 15 | Resident |
| Blacky^†^→Fanny | Bla | F | Adult | 49 | Resident |
| Nicky→Digit | Nic | F | Adult | 31 | Resident |
| Brigitte | Bri | F | Adult | 26 | Immigrant |
| Chicca^†^→Tzippi | Chi | F | Adult | 23 | Immigrant |
| Tzippi^†^ | Tzi | F | Adult | 15 | Immigrant |
| Balima | Bal | F | Adult | 11 | Resident |
| Elisha^†^ | Eli | F | Adolescent | 8 | Resident |
| Fanny | Fan | F | Adolescent | 7 | Resident |
