# Supplementary material for: Impartial Third-Party Interventions in Captive Chimpanzees: A Reflection of Community Concern
Source: PLoS One. 2012 Mar 7;7(3):e32494. doi: 10.1371/journal.pone.0032494 (PMC3296710; doi:10.1371/journal.pone.0032494)
Supplement: Table S2 — Ethogram used for observations during this study. (DOC) [file pone.0032494.s002.doc]

| **Abbreviation** | **Behaviour/Vocalization** | **Description of behaviour/vocalization** |
| --- | --- | --- |
| ***Affiliative behaviour*** |  |  |
| ap | Approach | An individual walks towards and stops within 2 metres of another individual. |
| bp | Look at | An individual looks at another individual or at an object (not necessarily affiliative). |
| fol | Follow | An individual keeps close behind another individual or moves along some distance behind it (along the same route) with its behaviour clearly oriented towards the individual. |
| fol-i | Invitation to follow | An individual walks in one direction, stopping and looking backward toward a partner (e.g. by a male to induce a sexual partner to follow). |
| pg | Allo-grooming | An individual parts another individual’s hair with its hands and picks up particles with either fingers or mouth. |
| pg2 | Mutual grooming | Individuals simultaneously part each other’s hair with their hands and pick up particles with either fingers or mouth |
| va | Pant | Fast inhalation/exhalation of air accompanied with quick rhythmic movements of the body |
| slp | Lip-smack | Almost exclusively occurring during grooming. Mouth is slightly opened and closed rhythmically. There is a smacking sound as the mouth is opened. |
| ps | Inspect genitals/anal region | An individual’s lips/fingers touch the genitals or anal region of another individual. Sometimes fingers are also inserted into these body parts. |
| pb | Hunch over | An individual stands on its feet, shoulders hunched up, arms held forward over the back of another individual. |
| pe | Embrace | An individual puts one or both arms around another individual. The other individual may do the same. An embrace can be performed ventro-ventrally or dorso-ventrally. |
| pr | Anti-parallel-ceremony | Individuals stand in body contact and face opposite directions. |
| ph | Touch | An individual reaches out with hand/foot and touches another individual. |
| pj | Hold hands | Individuals hold each other’s hand. |
| pa | Beg | Individual reaches out with hand (palmar surface faces upwards) in order to get something (e.g. food, social support) |
| p5 | Hand in mouth | An individual takes another individual’s hand/wrist into its mouth. |
| p6 | Finger in mouth | An individual takes another individual’s fingers into its mouth. |
| pm | Muzzle | An individual bites another individual in a playful manner. Mouth is pressed against the body of the other individual. |
| pk | Kiss | An individual kisses another individual. Mouth is pressed against the other’s mouth or nose is pressed against the other’s nose. |
| grasp | Grasping | An individual gently grasps any body part of another individual with one or both hands. |
| ***Play*** |  |  |
| sk | Play-wrestle | Two ore more individuals wrestle in a playful manner with each other (with body contact). |
| sj | Play-chase | Two or more individuals chase each other in a playful manner (without body contact). |
| tr | Play-face | Occurs during play. Individual retracts lip corners. Jaw may be open or closed. Teeth can be visible. |
| va | Pant | See above |
| vl | Laugh | Vocalization that resembles human laughter, but more throaty and hoarse. |
| ***Agonistic behaviour*** |  |  |
| *1) Quasi-aggression* |  |  |
| qa | Quasi-aggression, Tease | An individual teases another individual by spitting/throwing dirt at another individual. Sometimes also involves slapping. |
| b1 | Beginning bluff | An individual sways from one foot to the other (often in an upright position) or stamps the ground. Hair is often erected. |
| vu | Low hoot | Continuous low-pitched vocalizations given only by aroused individuals (without climax). |
| vo | Rising hoot | Continuous low-pitched vocalizations given only by aroused individuals (with climax). |
| ba | Charging display | An individual directs a feint attack towards another individual (without additional aggression like biting or hitting). |
| br | Run over | An individual jumps over another individual (without trampling the other individual). |
| upb | Undirected partial bluff | An individual sways from one foot to the other (hair often erected) and then runs towards an object hitting it with hand/feet (without giving a rising hoot). |
| ufb | Undirected full bluff | An individual sways from one foot to the other (hair often erected) and then runs towards an object hitting it with hand/feet (with giving a rising hoot). |
| ***2) Aggression*** |  |  |
| a1 | Aggression without body contact | An individual threatens another individual. Often involves screaming at another individual. |
| a2 | Aggression with body contact | An individual attacks another individual by at least beating it. |
| a3 | Severe aggression with body contact | An individual attacks another individual by hitting/ kicking it. Biting occurs and often wounds are inflicted. |
| a4 | Severe aggression with body contact (longer than 3 minutes) | Self-explanatory (see above) |
| vq | Scream | Vocalization given in order to threat another individual. |
| aj | Chase | An individual runs after another individual in aggression. |
| ad | Displace | An individual takes over the place of another individual. |
| vb | Waa bark | Loud, sharp vocalization given in a variety of agonistic contexts. |
| ***Submissive behaviour*** |  |  |
| vg | Pant grunt | Soft or loud vocalization (often emitted in a series) functioning as a signal of respect. |
| vk | Pant scream | Pant grunt that turns into a pant scream if the subordinate’s tension is high. |
| zb | Bow | An individual flexes its limbs so that the head or upper part of the body is lowered towards the ground. |
| tb | Silent bared teeth | An individual retracts its lip corners and shows its teeth, which are closed. |
| vs | Scream | Vocalization given when another individual or object scares an individual. |
| zp | Ano-genital presenting | An individual presents its ano-genital region towards another individual. |
| za | Avoid | An individual moves away in response to an approaching individual (unhurried). |
| zf | Flee | An individual rapidly moves away from another individual (e.g. after aggression received) or object. |
| ***Sexual behaviour*** |  |  |
| ef | Female presents | A female individual standing in front of a male individual with her swollen genitals turned towards him. |
| em | Male presents | A male individual shows his erected penis to a female individual (often in a sitting position). |
| ei | Inspect | A male individual sniffs/touches the swollen genitals of a female individual. Sometimes fingers are inserted into the genitals. |
| ep | Copulate | A male individual mounts (from behind) a swollen female individual and shows rhythmic dorso-ventral movements of the pelvis. |
| es | Masturbate | Self-manipulation of genital organs |
|  |  |  |
| *Alarm* |  |  |
| vw | Waow bark | The alarm call of chimpanzees |
| ***Other behaviours*** |  |  |
| yaw | Yawning | Self-explanatory |
| scr | Scratching | Individual uses hands/feet to scratch itself. |
| vf | Food grunts | Vocalizations uttered when expecting, seeing or approaching food. |
| le | Leave | An individual walks away from another individual and increases the distance to more than 2 metres. |
| tt | Temper tantrum | The chimpanzee way of expressing frustration, resembling that of human infants. |
| bp | Look at | An individual looks at another individual or at an object (not necessarily affiliative). |
| whp | Whimper | Soft vocalization (often emitted in a series) functioning to express frustration (often uttered by infants). |
| walk | Walk around | An individual walks on the substrate (e.g. ground, trees). |
| ***Tension*** |  |  |
| h0 | Stereotyping | E.g. an individual is rocking back and forth or plugs own hair extensively. |
| h1 | Auto-aggression | An individual hits itself. |
| dp | Piloerection | An individual bristles its hair. |
| er | Erection | A male individual has an erected penis. |
| ***3^rd^ party-interactions*** |  |  |
| AA | Support aggressor | An individual stands close behind or beside the aggressor and may attack or threaten the aggressee. |
| AU | Support aggressee | An individual stands close behind or beside the aggressee and may attack or threaten the aggressor. |
| UU | Impartial intervention | An individual breaks up a conflict without taking sides by e.g. attendance (the individual approaches the conflict and looks at the opponents without showing any affiliative or agonistic behaviours), interposition or running through the conflict. |
| FF | Protect, appease | An individual approaches another highly aroused individual (e.g. an aggressor) and directs affiliative or submissive behaviours at this individual in order to prevent (further) aggression. |
| TÖ | Console | After aggression occurred a third individual initiates affiliative contact with the recipient of aggression. |
| PÖ | Reconcile | Former opponents engage in affiliative behaviours with each other (e.g. embracing, kissing each other). |
| ***Proximity*** |  |  |
| ww | > 5 metres | An individual is at a distance of more than 5 metres from another individual |
| p0 | < 5 metres | An individual is within 5 metres of another individual. |
| p1 | Within 2x arm’s reach | An individual is within 2x an arm’s reach (of that individual) of another individual. |
| p2 | Within arm's reach | An individual is within an arm’s reach (of that individual) of another individual. |
| p4 | Huddle | Individuals actively huddle against each other. |
| p3 | Body contact | Individuals sit, stand or lay in a way that they touch each other without performing any of the other behaviours. |
| --2 | Mutual | Individuals engage in a given behaviour simultaneously (e.g. grooming). |
