# Supplementary material for: Impartial Third-Party Interventions in Captive Chimpanzees: A Reflection of Community Concern
Source: PLoS One. 2012 Mar 7;7(3):e32494. doi: 10.1371/journal.pone.0032494 (PMC3296710; doi:10.1371/journal.pone.0032494)
Supplement: Table S3 — Factors used in the generalized linear mixed models (GLMMs) to explain impartial interventions. a Directionality: scored as bidirectional if both participants engaged in aggressive behaviour and as unidirectional if all aggressive behaviour was directed toward the initial recipient. b Intensity: scored on a two-level scale: low intensity = conflict involved aggression without physical contact; high intensity = conflict involved physical aggression. c Complexity: scored as dyadic if one individual threatened or aggressed a second individual and as polyadic if more than two individuals were involved in the conflict. (DOC) [file pone.0032494.s003.doc]

| **Name** | **Type** |
| --- | --- |
| *Dependent variable* |  |
| Impartial intervention | Dichotomous (yes, no) |
|  |  |
| *Fixed explanatory factor (conflict characteristics)* |  |
| Directionality^a^ | Dichotomous (bi-, unidirectional) |
| Intensity^b^ | Dichotomous (high, low) |
| Complexity^c^ | Dichotomous (polyadic, dyadic) |
|  |  |
| *Fixed explanatory factor (conflict participants)* |  |
| Maternal kin | Dichotomous (yes, no) |
| “Friend” | Nominal (no, including one, including two) |
| Immigrant female | Dichotomous (yes, no) |
|  |  |
| *Fixed explanatory factor (class of conflict)* |  |
| Sex-dyad combination | Nominal (female-female, male-female, male-male) |
|  |  |
| *Fixed explanatory factor (identity of arbitrator)* |  |
| Male | Dichotomous (Dig, Ces) |
|  |  |
| *Random factor* |  |
| Date of data collection | Time |
